# Postintubation Tracheal Rupture in a Patient With Herpes Simplex Virus Type 1 (HSV-1) Tracheitis

**DOI:** 10.7759/cureus.71536

**Published:** 2024-10-15

**Authors:** Metri Haddaden, Louay H Aldabain, Dina Shaban, Ranim Bittar, Houssein Youness

**Affiliations:** 1 Pulmonary and Critical Care, University of Oklahoma Health Sciences Center, Oklahoma, USA; 2 Hospital Medicine, MedStar Good Samaritan Hospital, Baltimore, USA; 3 Internal Medicine, Michigan State University College of Human Medicine, Lansing, USA; 4 Pathology, University of Oklahoma Health Sciences Center, Oklahoma, USA

**Keywords:** herpes simplex-1 (hsv-1), peri-endobronchial tube irregular growth, post-intubation complications, tracheal injury, tracheal rupture

## Abstract

Herpes simplex virus type 1 (HSV-1) can cause a range of infections, including orolabial herpes and, in rare cases, tracheobronchitis, especially in immunocompromised patients. Inflammation from such infections might increase the risk of postintubation tracheal rupture, a serious complication characterized by subcutaneous and mediastinal emphysema and pneumothorax. This case report presents a rare instance of tracheal rupture following intubation in a patient with HSV-1 tracheitis.

A 70-year-old woman with diabetes mellitus presented to the hospital with altered mental status after drinking a bottle of Coke. She was diagnosed with diabetic ketoacidosis, and multifocal pneumonia which were treated with insulin, antibiotics, and supportive care. She was also intubated for airway protection. After a few days of treatment, her mental status and medical condition improved warranting extubation. Post-extubation, she developed stridor that did not respond to medical treatment. In concerns for airway compromise, an attempt of re-intubation was complicated with hypoxemia, subcutaneous face and chest emphysema, pneumoperitoneum, and bilateral pneumothoraces leading to cardiopulmonary arrest. Cardiopulmonary resuscitation was started, and return of spontaneous circulation was achieved after one minute. She was eventually intubated with a smaller endotracheal tube, and bilateral chest tubes were placed. Bronchoscopy revealed tracheal injury and whitish growth around the endotracheal tube. A biopsy of the latter growth was consistent with HSV-1 infection, prompting antiviral therapy. The patient ultimately recovered and was discharged after tracheostomy placement.

This case represents a rare instance of iatrogenic tracheal injury associated with HSV-1 tracheitis. Diagnosis of tracheal injury hinges on clinical suspicion and is confirmed through radiologic imaging and bronchoscopy. Treatment varies based on injury severity, with our patient's level II injury managed conservatively with antiviral therapy and tracheostomy, resulting in a favorable outcome.

## Introduction

Herpes simplex virus type 1 (HSV-1) is a linear double-stranded deoxyribonucleic acid (dsDNA) with an icosahedral capsid and spikey envelope [1]. It is a member of the Herpesviridae family [2]. HSV-1 is frequently asymptomatic but could present itself as orolabial herpes, herpetic sycosis, herpes gladiatorum, herpetic whitlow, ocular HSV infection, encephalitis, and Kaposi varicelliform eruption [1]. It could also rarely cause tracheobronchitis particularly in the immunocompromised population [3]. Inflammatory conditions affecting the tracheobronchial tree are known to increase the risk of postintubation tracheal rupture [4]. The latter is a rare but life-threatening complication with a roughly estimated incidence of 0.05% to 0.37% of all orotracheal intubations performed [4]. The clinical manifestations of postintubation tracheal rupture are subcutaneous emphysema, mediastinal emphysema, pneumothorax along with dyspnea, dysphonia, cough, hemoptysis, and pneumoperitoneum [4]. The symptomatology of this rare complication happens immediately, soon after extubation, or sometimes takes a few days to appear [4]. Here, we report a very rare case of tracheal rupture following orotracheal intubation in a patient with HSV-1 tracheitis.

## Case presentation

A 70-year-old woman with a past medical history of diabetes mellitus presented initially to Lindsay Municipal Hospital with altered mental status. A few hours before her presentation, she reported fatigue and consumed a bottle of Coke. Shortly after, she became altered and incoherent. Upon arrival at Lindsay Municipal Hospital, she was confused and exhibited signs of seizure activity for which she received lorazepam and midazolam. She was also hypertensive and tachycardic with a blood glucose exceeding 800 mg/dL. Due to concerns for airway protection, endotracheal intubation was attempted but proved unsuccessful. Ultimately, a laryngeal mask airway was placed and was transferred to the University of Oklahoma Medical Center (OUMC) for further management.

Upon arrival at our hospital, her Glasgow coma scale (GCS) was 6 and was intubated successfully. Vital signs revealed a temperature of 35.5°C, a heart rate of 117 beats per minute, and a blood pressure of 129/88 millimeters of mercury (mmHg). Physical exam showed coarse bronchial breathing bilaterally and faint heart sounds. Laboratory results were significant for a hemoglobin level of 11 g/dL, white blood count of 4.69 K/cmm, sodium of 138 mmol/L, potassium of 5.5 mmol/L, bicarbonate of 14 mmol/L, anion gap of 18, glucose of 740 mg/dL, creatinine of 1.89 mg/dL, pro B-type natriuretic peptide of 212 pg/L, and a peak troponin level of 67 ng/mL. Other notable results included a lactic acid level of 3.8 mmol/L, glucosuria on urine analysis, and a venous blood gas showing a pH of 7.19.

Chest X-ray revealed bilateral pulmonary opacities. Chest computed tomography (CT) without contrast demonstrated bilateral diffuse consolidative and ground-glass opacities consistent with multifocal pneumonia. The patient was started on a vancomycin, ampicillin-sulbactam, doxycycline, isotonic intravenous (IV) fluid, norepinephrine to maintain mean arterial pressure above 65 mmHg, and insulin drip for diabetic ketoacidosis. Bronchoscopy with bronchoalveolar lavage (BAL) was performed on the day of admission and showed thick secretions with no bacterial culture growth. Her antibiotics were then changed to piperacillin-sulbactam and vancomycin and later only piperacillin-tazobactam was continued.

Infectious workup including respiratory virus panel, methicillin-resistant *Staphylococcus aureus* (MRSA), urine streptococcus and legionella antigens, tracheal aspirate cultures, BAL cultures, blood cultures, beta-D-glucan, histoplasma urine antigen, cryptococcal serum antigen, *Bordetella pertussis *and parapertussis, mycoplasma, and *Chlamydia pneumoniae *polymerase chain reaction (PCR) were all negative. 

**Figure 1:**
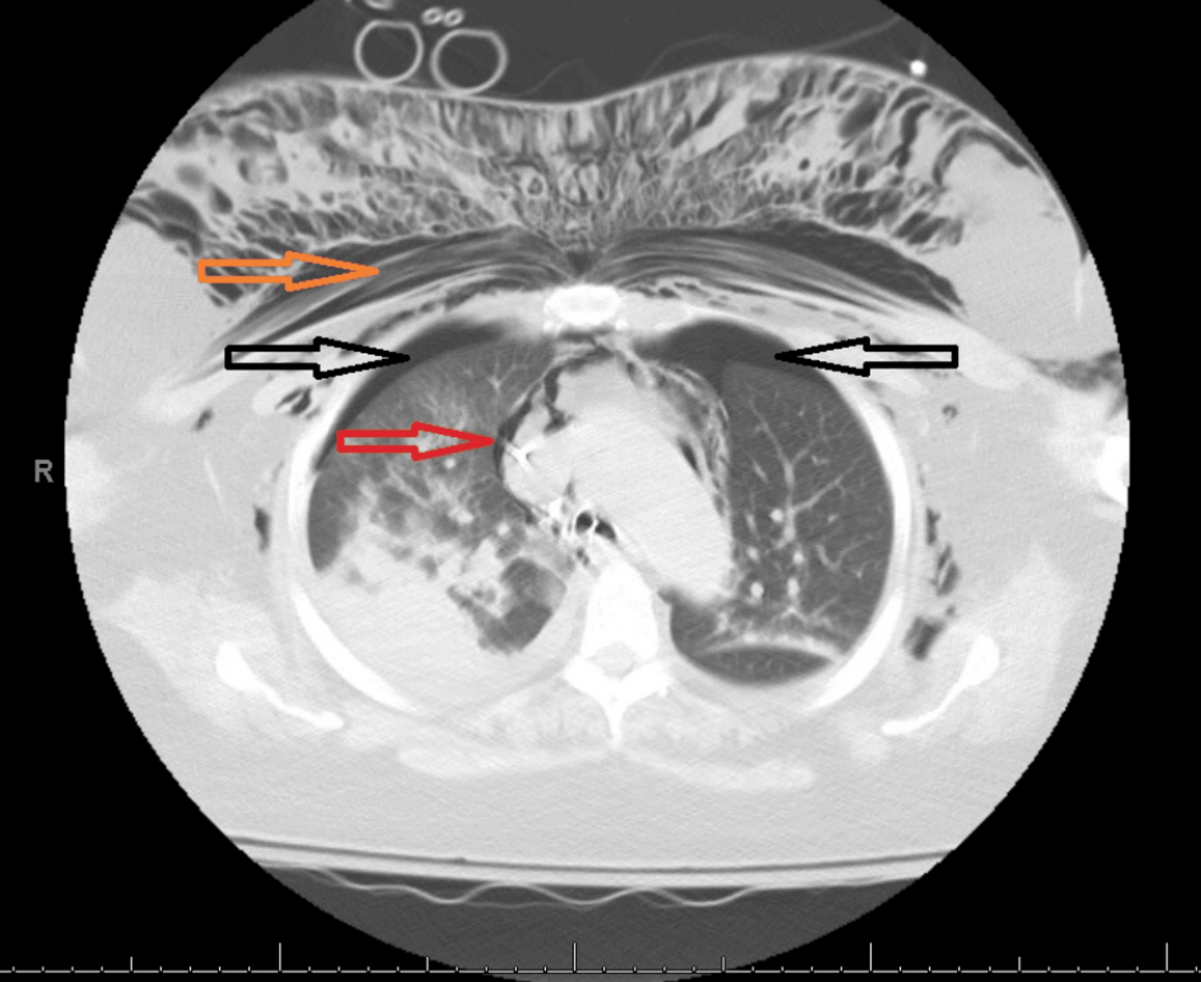
CT chest showing bilateral pneumothroaces (black arrows), pneumomediastinum (red arrow), and subcutaneous emphysema (orange arrow)

On day 5, the patient improved and was extubated to bi-level positive airway pressure (BiPAP) ventilation. Post-extubation, she developed stridor, prompting the administration of methylprednisolone IV (20 mg) and nebulized racemic epinephrine twice with no improvement. Due to concerns for upper airway compromise, reintubation was attempted with 7.5 mm but was unsuccessful. On the second attempt, the endotracheal tube (ETT) was successfully passed through the vocal cords but faced resistance with no color change in capnography upon ventilation. Suddenly, her face and chest developed subcutaneous emphysema. The ETT was removed, and bag mask ventilation was started for hypoxemia but failed to improve her oxygenation. She then became more hypoxemic and hypotensive and went into pulseless electrical activity (PEA) arrest. Advanced life support cardiopulmonary resuscitation was started, and return of spontaneous circulation was achieved after one minute. She was subsequently intubated with 6 mm ETT. CT chest showed subcutaneous emphysema, bilateral pneumothoraces, and pneumomediastinum (Figure 1).

**Figure 2:**
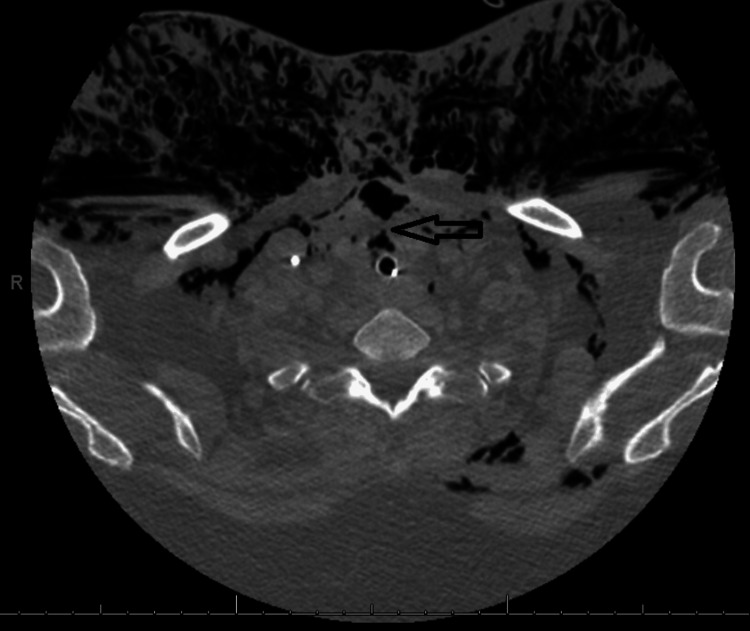
CT neck showing possible focal defect in the anterior tracheal wall (black arrow)

CT neck showed a possible small focal defect in the anterior tracheal wall about 1.5 cm below the cricoid cartilage (Figure 2).

Bilateral chest tubes were inserted and left to suction -20 mmHg, steroids were given for airway edema, and antimicrobial drugs were started; the latter was changed based on clinical suspicion and culture and sensitivity. Her tracheal aspirate culture ended up growing *Citrobacter *and *Klebsiella *which was covered with cefepime.

Bronchoscopies on day 5, 8, and 9, aided by simultaneous video-assisted laryngoscopies, revealed the ETT beyond the tracheal injury. The ETT was retracted under bronchoscopic guidance to evaluate the subglottic area and an area of stenosis with irregular whitish tissue growth were seen. Attempts to advance the bronchoscopes through the vocal cords were hindered by edema, accompanied by audible stridor heard upon ETT cuff deflation.

**Figure 3:**
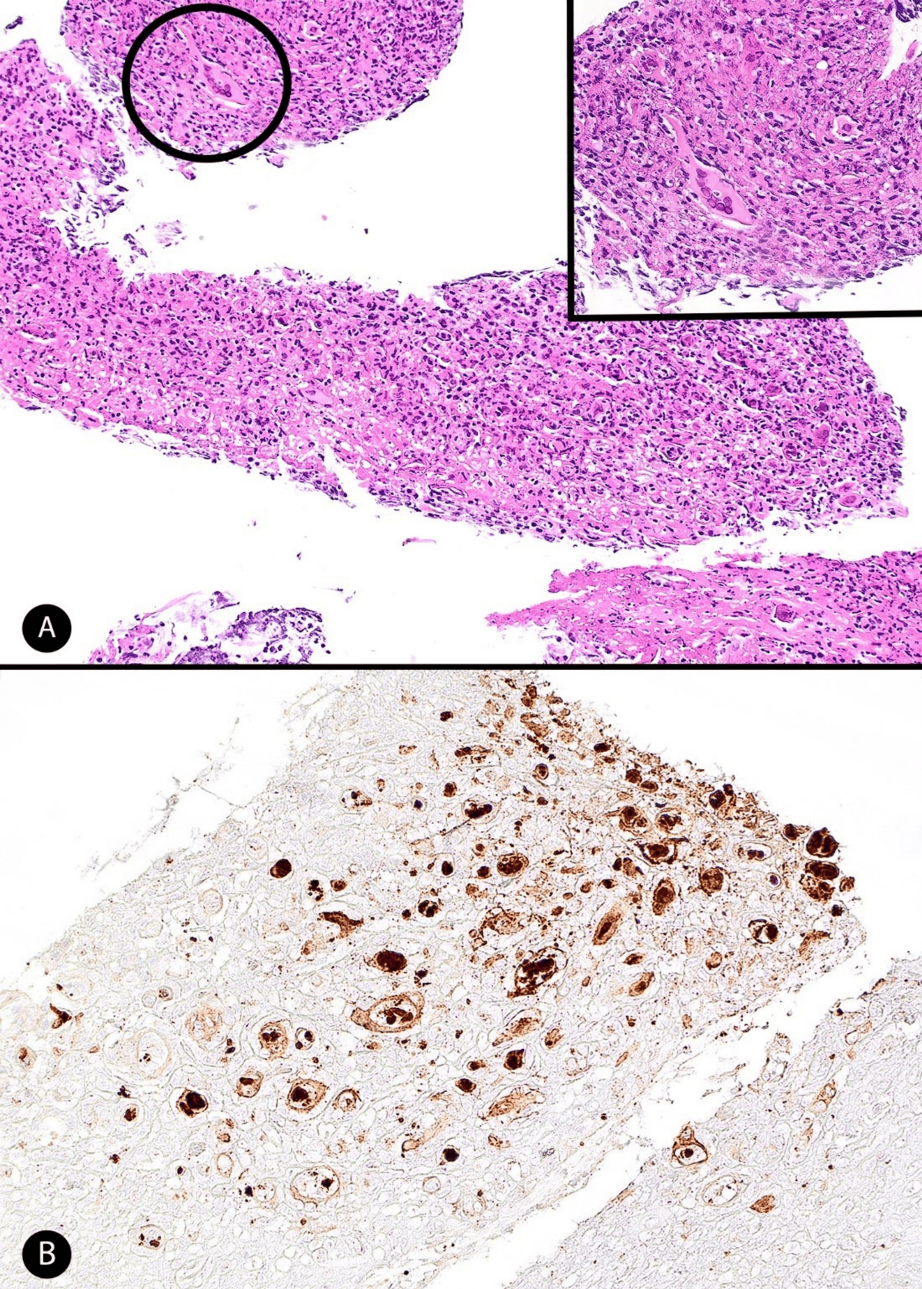
Tracheal biopsy HSV: Herpes simplex virus (A) Section of the tracheal biopsy showing inflammatory debris, fibrin, and desquamated epithelial cells (hematoxylin and eosin (H&E), 10X magnification). Inset: Higher magnification of the epithelial cells showing viral cytopathic effects characteristics of HSV, including multinucleated giant cells with nuclear molding, prominent ground-glass intranuclear inclusions, and a rim of condensed chromatin (H&E, 20X magnification). (B) HSV1/2 immunohistochemical stain showing strong nuclear and cytoplasmic positivity in virally infected cells, confirming the presence of HSV

On day 11, a pediatric bronchoscope identified false vocal cord swelling, peri-ETT subglottic irregular whitish tissue growth and significant airway narrowing, preventing passage between the ETT cuff and the trachea. Tissue biopsy from the irregular tissue growth and BAL were obtained, revealing neutrophilic fibrin exudate, desquamated epithelial cells including epithelial cells with HSV viral cytopathy (Figure 3).

BAL was positive for HSV-1, *Citrobacter*, and *Klebsiella*. Acyclovir was initiated, subsequently switched to valacyclovir, and cefepime was replaced with ciprofloxacin based on the infectious disease team recommendations. Ultimately, tracheostomy was placed, and chest tubes were removed. She was discharged after almost a month and half in the hospital. 

## Discussion

Multiple risk factors have been identified, including but not limited to older age, female gender, obesity, prolonged use of steroids, local inflammation, anatomical variations or changes like tracheal diverticula or neoplasms, presence of neck or mediastinal masses causing tracheal displacement, pronounced cervical lordosis or scoliosis, and a history of multiple intubation attempts or limited experience in procedural performance [5]. We identified female sex, patient’s age, multiple intubations, and HSV-1 tracheitis as risk factors in our case. We hypothesize that her severe hyperglycemia led her to be immunocompromised, increasing her risk for HSV activation which led to local inflammation and stenosis. Throughout our literature search, we haven’t found any reported cases of iatrogenic tracheobronchial injury associated with HSV-1 tracheitis, making our case the first to be reported.

Diagnosis of tracheobronchial injury primarily depends on clinical suspicion. Radiologic imaging using chest X-ray or CT scan can aid in diagnosing and characterizing the lesion with the latter being more sensitive and accurate [5]. The addition of contrast to CT scan enhances the ability to detect the injury itself and any other damage to mediastinal organs [5]. While imaging yields valuable information, the gold standard for diagnosis still lies in endoscopic imaging and should be pursued in a timely manner [5].

Cardillo et al. (2010)* *categorized tracheal injuries based on the severity of the damage. A level I injury involves mucosal or submucosal tracheal involvement without mediastinal emphysema and esophageal injury. In level II injuries, there is a tracheal lesion extending up to the muscular wall, accompanied by subcutaneous or mediastinal emphysema but without esophageal injury or mediastinitis. Level IIIA refers to a complete laceration of the tracheal wall with esophageal or mediastinal soft-tissue hernia, excluding esophageal injury or mediastinitis. Lastly, level IIIB encompasses any tracheal wall laceration associated with esophageal injury or mediastinitis [6].

Traditionally, surgical repair has been the gold standard of care for postintubation tracheobronchial injury [7]. Nowadays, other therapeutic modalities have emerged, including conservative and endoscopic treatments [5]. However, we still lack definitive guidelines to standardize intervention, making treatment more personalized on a case-by-case basis, depending on the characteristics of injury, the patient’s condition, and the experience of the center [5].

Conservative management is usually pursued in asymptomatic patients with level I injuries, and its use has been expanding to include more severe injuries reaching up to level IIIA tears. Conservative management harbors options that include observation, intubation, tracheostomy, and fibrin glue application. Endoscopic management can be used to treat patients with level IIIA and some IIIB level injuries. It is preferable to reserve surgical intervention for highly symptomatic large IIIA level injuries especially if mechanical ventilation is ineffective and for level IIIB injuries. In concordance with available literature, she was managed conservatively with acyclovir and continued endotracheal intubation, with tracheostomy tube placement.

## Conclusions

This case underscores the complexity of iatrogenic tracheal rupture diagnosis and management. The patient's advanced age, multiple intubation attempts, and immunocompromised state due to severe hyperglycemia were significant risk factors leading to tracheal injury. We also hypothesize that HSV-1 tracheitis made the trachea more prone to such injury. Prompt diagnosis through imaging and endoscopic evaluation, combined with a tailored conservative management approach, resulted in a favorable outcome in our patient. This case adds to the limited body of literature on HSV-1-associated iatrogenic tracheal injuries. More research is needed to prove this association.
